# RETRACTED: Chen et al. A Novel Drug Self-Delivery System from Fatty Alcohol Esters of Tranexamic Acid for Venous Malformation Sclerotherapy. *Pharmaceutics* 2022, *14*, 343

**DOI:** 10.3390/pharmaceutics16020199

**Published:** 2024-01-30

**Authors:** Yongfeng Chen, Di Song, Qianqian Hou, Mengrui Ma, Xiaoyun Zhao, Tianzhi Yang, Huichao Xie, Pingtian Ding

**Affiliations:** 1 School of Pharmacy, Shenyang Pharmaceutical University, Shenyang 110016, China; 2 School of Life Science and Biopharmaceutics, Shenyang Pharmaceutical University, Shenyang 110016, China; 3 Department of Basic Pharmaceutical Sciences, School of Pharmacy, Husson University, Bangor, ME 13802, USA; yangt@husson.edu

The Pharmaceutics Editorial Office retracts the article, “A Novel Drug Self-Delivery System from Fatty Alcohol Esters of Tranexamic Acid for Venous Malformation Sclerotherapy” [1], cited above.

Following publication, the authors raised concerns to the publisher regarding a number of scientific and images errors.

Adhering to our complaint’s procedure, an investigation was conducted by the Editorial Office and Editorial Board, that confirmed the overlap of subsections of Figure 7B (NS group, 1 d and 15 d), incorrect association of subfigures 8B and 8C to the named experimental animals, and writing errors in the Material and Method section. 

As a result, the Editorial Office and Editorial Board were unable to confirm the reliability of the findings and subsequently decided to retract this paper as per MDPI’s retraction policy (https://www.mdpi.com/ethics#_bookmark30). 

This retraction was approved by the Editor-in-Chief of the journal *Pharmaceutics*.

The authors agreed to this retraction.

## References

[1] Chen Y., Song D., Hou Q., Ma M., Zhao X., Yang T., Xie H., Ding P. (2022). RETRACTED: A Novel Drug Self-Delivery System from Fatty Alcohol Esters of Tranexamic Acid for Venous Malformation Sclerotherapy. Pharmaceutics.

